# Determination of Reference microRNAs for Relative Quantification in Porcine Tissues

**DOI:** 10.1371/journal.pone.0044413

**Published:** 2012-09-10

**Authors:** Oriol Timoneda, Ingrid Balcells, Sarai Córdoba, Anna Castelló, Armand Sánchez

**Affiliations:** Departament de Genètica Animal, Centre de Recerca en AgriGenòmica (CRAG), Universitat Autònoma de Barcelona (UAB), Bellaterra, Barcelona, Spain; University of Minnesota Medical School, United States of America

## Abstract

Relative quantification is the strategy of choice for processing RT-qPCR data in microRNAs (miRNAs) expression studies. Normalisation of relative quantification data is performed by using reference genes. In livestock species, such as pigs, the determination of reference miRNAs and the optimal number of them has not been widely studied. In this study, the stability of ten miRNAs (Ssc-let-7a, Ssc-miR-103, Ssc-miR-17-3p, Hsa-miR-25, Hsa-miR-93, Ssc-miR-106a, Ssc-miR-191, Ssc-miR-16, Ssc-miR-26a and Ssc-miR-17-5p) was investigated by RT-qPCR in different tissues (skeletal muscle, kidney, liver, ovary and uterus) and in different pig breeds (Iberian, Landrace, Large White, Meishan and Vietnamese) as variation factors. Stability values were calculated with geNorm and NormFinder algorithms obtaining high correlation between them (r^2^ = 0.99). The analyses showed that tissue is an important variability factor in miRNAs expression stability whereas breed is not a determinant factor. All ten miRNAs analysed had good stability values and, therefore, can be used as reference miRNAs. When all tissues were considered, miR-93 was the most stable miRNA. Dividing data set by tissues, let-7a was the most stable in skeletal muscle and ovary, miR-17-5p in kidney, miR-26a in liver and miR-103 in uterus. Moreover, the optimal number of reference miRNAs to be used for proper normalisation data was determined. It is suggested the use of five reference miRNAs (miR-93, miR-25, miR-106a, miR-17-5p and miR-26a) in multi-tissue experimental designs and the use of three reference miRNAs as the optimal number in single tissues studies (let-7a, miR-17-5p and miR-25 in skeletal muscle; miR-17-5p, miR-93 and miR-26a in kidney, miR-26a, miR-103 and let-7a in liver, let-7a, miR-25 and miR-106a in ovary and miR-103, let-7a and miR-93 in uterus). Overall, this study provides valuable information about the porcine reference miRNAs that can be used in order to perform a proper normalisation when relative quantification by RT-qPCR studies is undertaken.

## Introduction

MicroRNAs (miRNAs) are small non-coding RNAs involved in gene expression regulation at the post-transcriptional level in animals, plants and viruses [1]–[3]. They participate in a wide range of biological processes where they play important roles. Largely, their role involves blocking protein translation and/or inducing mRNA degradation [4]. Moreover, miRNA expression has been associated with different pathological processes, such as cancer, neurological disorders, inflammatory pathologies and cardiovascular diseases [5]–[8]. In some of these pathologies, it has been suggested that miRNAs can be used as biomarkers to develop new diagnostic tools [9], [10]. Therefore, it is very important to measure the miRNA expression with high accuracy.

Northern blot has been widely used for determining and measuring miRNA expression [11]. However, latest approaches such as DNA chips (microarrays), high-throughput sequencing (HTS) and reverse transcription quantitative real-time polymerase chain reaction (RT-qPCR) are also commonly used [12], [13]. HTS and microarrays are used to determine miRNA expression at a genome-wide level whereas RT-qPCR is used to measure the expression of a specific miRNA [14], [15]. Furthermore, RT-qPCR is used to validate expression studies done by microarrays and by HTS due to its high sensitivity and reproducibility [16]. Thus, RT-qPCR has become an important method to assess miRNA expression.

One of the most extensive strategies used to evaluate and compare RT-qPCR data is relative quantification [17]. This methodology normalises the expression of the genes of interest by using one or more genes, called reference genes, of which expression is stable. Data normalisation is necessary to control variables like equal mass loading which can introduce false differences in expression and can perform some experimental bias in the results. Moreover, it is essential to control other variation factors such as RNA degradation during sample processing, quality differences between samples, initial concentration variation among samples, technical variations like pipetting errors and other factors which can affect accuracy during the technique processing [18]. For this reason it is mandatory to perform a data normalisation strategy to correct these possible biases. Originally, normalisation strategies were performed using only one reference gene. However, this idea has evolved to different normalisation approximations, from using the global mean normalisation method [19] to the robust multiple reference genes normalisation method [17] where more than one reference gene is used. The reference genes chosen (acting as endogenous controls) must not be affected by experimental parameters and they must show invariant expression to the exposed conditions of the individuals used in the study. Consequently, reference genes, as stable genes, are generally involved in basic cellular processes.

In miRNA expression studies, the most common reference genes used are ribosomal RNAs, such as 5S RNA [20]–[22] and small nuclear RNAs like RNU6B [10], [19]–[24]. However, the use of miRNAs as reference genes is still not widely used; although it is very important that the references used have the same nature that the study subjects. The reference genes used should have the same length as the molecules of interest in order to assure the same efficiency during RNA isolation and reverse transcription [25]. In this sense, only a few studies have explored the stability of some miRNAs in human tissues [19]–[21] and the published works are largely related to cancer processes [24]–[28]. Focusing on miRNA expression studies in livestock species, there are few works using miRNAs as reference control in miRNA expression analysis by relative quantification [29]–[31] and only one report has deeply analysed the miRNA expression stability in pigs to be used as reference miRNAs [22].

The aim of this study was to analyse the miRNA expression stability in different porcine tissues and breeds. Selected tissues were skeletal muscle (structural tissue), uterus and ovary (reproductive tissues), liver (metabolic tissue) and kidney (excretory tissue). On the other hand, porcine breeds include Iberian (European breed), Meishan and Vietnamese (Asian breeds) and Landrace and Large White (European commercial breeds). The results from this work provide useful information concerning which miRNAs could be effectively used as reference genes in order to measure miRNA expression accurately through RT-qPCR studies.

## Results and Discussion

### Analysis of the Stability of the Reference miRNAs

In accordance with the most stable miRNAs described in the literature [20]–[22], [24], [26]–[28], ten candidate miRNAs (Ssc-let-7a, Ssc-miR-103, Ssc-miR-17-3p, Hsa-miR-25, Hsa-miR-93, Ssc-miR-106a, Ssc-miR-191, Ssc-miR-16, Ssc-miR-26a and Ssc-miR-17-5p, Table 1) were selected to study their expression stability in different porcine tissues and breeds. All candidate reference miRNAs were successfully amplified through RT-qPCR, allowing us to perform adequate genetic expression quantification [32]. Efficiencies obtained were high ranging from 90% to 110% and the standard curves correlations were at 0.995 minimum (Table 2).

**Table 1:** Primers and miRNA sequences used for the RT-qPCR design.

**miRNA**	**Sequence (5′-3′)**	**Forward primer (5′-3′)**	**Reverse primer (5′-3′)**
Ssc-let-7a	TGAGGTAGTAGGTTGTATAGTT	GCAGTGAGGTAGTAGGTTGT	GGTCCAGTTTTTTTTTTTTTTTAACTATAC
Ssc-miR-103	AGCAGCATTGTACAGGGCTATGA	AGAGCAGCATTGTACAGG	GGTCCAGTTTTTTTTTTTTTTTCATAG
Ssc-miR-17-3p	ACTGCAGTGAAGGCACTTGTAG	GACTGCAGTGAAGGCA	GTCCAGTTTTTTTTTTTTTTTCTACAAG
Hsa-miR-25	CATTGCACTTGTCTCGGTCTGA	CATTGCACTTGTCTCGGT	GGTCCAGTTTTTTTTTTTTTTTCAGA
Hsa-miR-93	CAAAGTGCTGTTCGTGCAGGTAG	GCAAAGTGCTGTTCGTG	TCCAGTTTTTTTTTTTTTTTCTACCT
Ssc-miR-106a	AAAAGTGCTTACAGTGCAGGTAGC	GAAAAGTGCTTACAGTGCAG	TCCAGTTTTTTTTTTTTTTTGCTAC
Ssc-miR-191	CAACGGAATCCCAAAAGCAGCTG	AACGGAATCCCAAAAGCA	TCCAGTTTTTTTTTTTTTTTCAGC
Ssc-miR-16	TAGCAGCACGTAAATATTGGCG	GCAGTAGCAGCACGTA	CAGTTTTTTTTTTTTTTTCGCCAA
Ssc-miR-26a	TTCAAGTAATCCAGGATAGGCT	GCAGTTCAAGTAATCCAGGA	TCCAGTTTTTTTTTTTTTTTAGCCT
Ssc-miR-17-5p	CAAAGTGCTTACAGTGCAGGTAG	CAAAGTGCTTACAGTGCAG	GGTCCAGTTTTTTTTTTTTTTTCTAC

**Table 2:** Summary of qPCR assays for each reference miRNA studied.

**Reference miRNA**	**Primer conc. (nM each)**	**cDNA dilution**	**qPCR efficiency mean** *	**Std. curve correlation mean** *
Ssc-let-7a	125	1/2000	95.52% (2.11%)	0.9991 (0.0005)
Ssc-miR-103	250	1/2000	96.27% (5.15%)	0.9981 (0.0013)
Ssc-miR-17-3p	250	1/200	99.96% (7.25%)	0.9971 (0.0026)
Hsa-miR-25	250	1/2000	97.14% (3.76%)	0.9989 (0.0004)
Hsa-miR-93	200	1/2000	98.10% (3.12%)	0.9973 (0.0012)
Ssc-miR-106a	250	1/2000	99.73% (11.05%)	0.9978 (0.0015)
Ssc-miR-191	250	1/2000	97.45% (3.91%)	0.9978 (0.0009)
Ssc-miR-16	250	1/2000	98.31% (4.98%)	0.9990 (0.0004)
Ssc-miR-26a	250	1/2000	93.35% (0.62%)	0.9991 (0.0005)
Ssc-miR-17-5p	250	1/2000	98.52% (5.00%)	0.9980 (0.0009)

* The numbers in brackets denote the standard error for the mean values.

**Figure 1:**
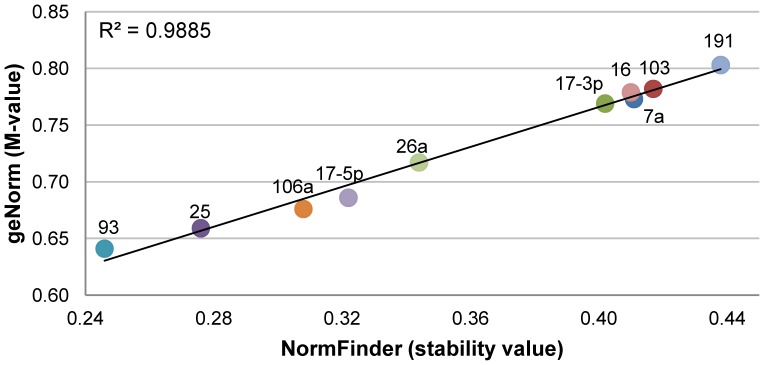
Correlation between M-value (geNorm^1^) and stability value (NormFinder^2^) in the general data set. ^1^: [17], ^2^: [33] A very good correlation between the two approximations confirms the robustness and the credibility of our results.

Firstly, the stability was evaluated taking into account the entire data (all tissues and pig breeds) with geNorm [17] and NormFinder [33] algorithms resulting from its correlation were in a good agreement (r^2^ = 0.99, Figure 1) and confirm the robustness of our results. GeNorm considers a putative reference gene when the M-value calculated is lower than 1.5 and NormFinder establishes a lower stability value indicating a better reference gene. All ten miRNAs evaluated had good stability values ranging from 0.64 to 0.80 in M Values (M, geNorm) and from 0.25 to 0.44 in stability values (SV, NormFinder). In this way, all analysed miRNAs can be used as reference miRNAs for miRNAs expression studies in pigs.

Although all miRNAs showed good stability values, the most stable miRNA was miR-93, followed by miR-25, miR-106a, miR-17-5p and miR-26a (Figure 1). Interestingly, these results are in accordance with a previous report in human tissues [20]. In contrast, miR-191 (M = 0.80, SV = 0.44), a common reference miRNA used in several human studies and one of the best reference miRNA in human tissues [20], [21], was determined as the least stable miRNA in pigs. Then, this result suggested that miR-191 expression stability depends on the specie studied. Comparing our data with a previous study performed also in pigs [22], there are some discordances. In our study, miR-103 was the second worst ranked whereas it was the best positioned in the study by [22]. Moreover, miR-106a (M = 0.68, SV = 0.31), a well ranked miRNA in our study, was the second least stable miRNA in [22] study. Despite of these discordances, miR-17-5p (M = 0.69, SV = 0.32) was well ranked in both studies and miR-16 (M = 0.78, SV = 0.41) had discrete M and SV values in the two studies. These variations in miRNA stability expression could be explained considering the differences of type and amount of tissues used in each study. The work performed by [22] used a total of 47 tissues where uterus was not included, but it was added in the present study. It is also important to remark that some referenced studies were from human tissues [20], [21] and it could also be a source of variation in miRNA stability expression when results are compared. Thus, it is reflected that before performing a RT-qPCR study, the reference genes used must be tested experimentally due to many influencing factors. In this sense, M-values obtained by [22] ranged from 1.0 to 2.3, considerably higher than our M-values that could also be explained by the difference in type and amount of tissues used in both studies.

It is known that the stability of miRNA expression could change when factors like tissue or breed are considered. Thus, the entire data were divided by breeds. The results obtained did not differ greatly compared to when the entire data were analysed. M and SV values were calculated from 0.64 to 0.90 and from 0.22 to 0.53, respectively (data not shown). The most stable miRNAs were still ranging between those most stable in the general study. miR-93 remained the most stable miRNA in Iberian and Meishan breeds, miR-26a in Landrace and Vietnamese breeds and miR-25 in Large White breed. The least stable miRNAs were miR-191 in Iberian and Landrace breeds, miR-16 in Large White and Vietnamese breeds and miR-26a in Meishan breed (data not shown). Overall, our results showed that breed only slightly influences the stability of miRNAs.

The stability of the miRNAs was also evaluated for each of the five tissues analysed. As expected, the stability of the miRNAs varies among tissues (Table 3). M and SV values were from 0.41 to 0.90 and from 0.11 to 0.57, respectively. Let-7a was the most stable miRNA in ovary and skeletal muscle, miR-103 in uterus, miR-17-5p in kidney and miR-26a in liver, evidencing the specificity of each tissue developing characteristic biological functions and specific metabolic pathways. Conversely, miR-16 was the least stable miRNA in kidney, uterus and liver, miR-103 in ovary and miR-17-3p in skeletal muscle. Stability values in skeletal muscle were in accordance with the results obtained by [22] in the porcine muscle-type tissue group. Two-way analysis of variance (ANOVA) including breed, tissue and tissue by breed interaction showed that only the tissue had a significant effect on miRNA expression (p-value <0.05) in all reference miRNAs analysed. However, in case of Ssc-miR-17-3p, breed and tissue by breed interaction had a significant effect on miRNA expression. These results show that tissue is an important variability factor that affects the stability miRNAs expression. Thus, it is evidenced the necessity of using reference miRNAs according the tissue analysed. However, although the high divergence between breeds originated by pig breeding, results showed that miRNA expression is stable across different breeds.

**Table 3:** Stability values for each microRNA calculated by using geNorm^a^ and NormFinder^b^ algorithms.

**Data set**	**Algorithm**	**miR-191**	**miR-106a**	**miR-25**	**miR-93**	**miR-17-5p**	**miR-26a**	**Let-7a**	**miR-103**	**miR-16**	**miR-17-3p**	**Correlation**
Kidney	gN	0.455^(7)^	0.452^(6)^	0.560^(8)^	0.417^(2)^	**0.410^(1)^**	0.430^(3)^	0.448^(5)^	0.446^(4)^	0.638^(10)^	0.616^(9)^	0.9952
	NF	0.206^(7)^	0.195^(6)^	0.319^(8)^	0.148^(2)^	**0.142^(1)^**	0.169^(3)^	0.195^(5)^	0.196^(4)^	0.392^(10)^	0.370^(9)^	
Ovary	gN	0.642^(9)^	0.523^(3)^	0.516^(2)^	0.576^(7)^	0.572^(6)^	0.580^(8)^	**0.500^(1)^**	0.667^(10)^	0.563^(4)^	0.571^(5)^	0.9897
	NF	0.359^(9)^	0.233^(3)^	0.228^(2)^	0.290^(7)^	0.295^(6)^	0.293^(8)^	**0.197^(1)^**	0.383^(10)^	0.265^(4)^	0.281^(5)^	
Uterus	gN	0.552^(4)^	0.593^(5)^	0.607^(6)^	0.544^(3)^	0.625^(7)^	0.648^(8)^	0.526^(2)^	**0.481^(1)^**	0.834^(10)^	0.712^(9)^	0.9714
	NF	0.219^(4)^	0.296^(5)^	0.294^(6)^	0.205^(3)^	0.337^(7)^	0.335^(8)^	0.190^(2)^	**0.106^(1)^**	0.520^(10)^	0.411^(9)^	
Skeletal	gN	0.581^(7)^	0.579^(6)^	0.529^(3)^	0.634^(8)^	0.516^(2)^	0.529^(4)^	**0.512^(1)^**	0.573^(5)^	0.714^(9)^	0.901^(10)^	0.9877
Muscle	NF	0.294^(7)^	0.267^(6)^	0.220^(3)^	0.319^(8)^	0.199^(2)^	0.233^(4)^	**0.182^(1)^**	0.271^(5)^	0.398^(9)^	0.566^(10)^	
Liver	gN	0.513^(5)^	0.528^(6)^	0.555^(9)^	0.510^(4)^	0.544^(7)^	**0.443^(1)^**	0.480^(3)^	0.456^(2)^	0.636^(10)^	0.549^(8)^	0.9825
	NF	0.257^(5)^	0.269^(6)^	0.293^(9)^	0.246^(4)^	0.293^(7)^	**0.169^(1)^**	0.209^(3)^	0.176^(2)^	0.367^(10)^	0.288^(8)^	
General	gN	0.803^(10)^	0.676^(3)^	0.659^(2)^	**0.641^(1)^**	0.686^(4)^	0.717^(5)^	0.773^(7)^	0.782^(9)^	0.779^(8)^	0.769^(6)^	0.9885
	NF	0.438^(10)^	0.308^(3)^	0.276^(2)^	**0.246^(1)^**	0.322^(4)^	0.344^(5)^	0.411^(7)^	0.417^(9)^	0.410^(8)^	0.402^(6)^	

a : [17], ^b^: [33].

gN: geNorm algorithm, NF: NormFinder algorithm. Correlations between M-value (geNorm) and stability value (NormFinder) are shown. Superscript numbers into brackets show stability values sorted out for each data set group from 1 (most stable) to 10 (less stable). The stability values of the most stable miRNA for each group are marked in bold.

**Figure 2:**
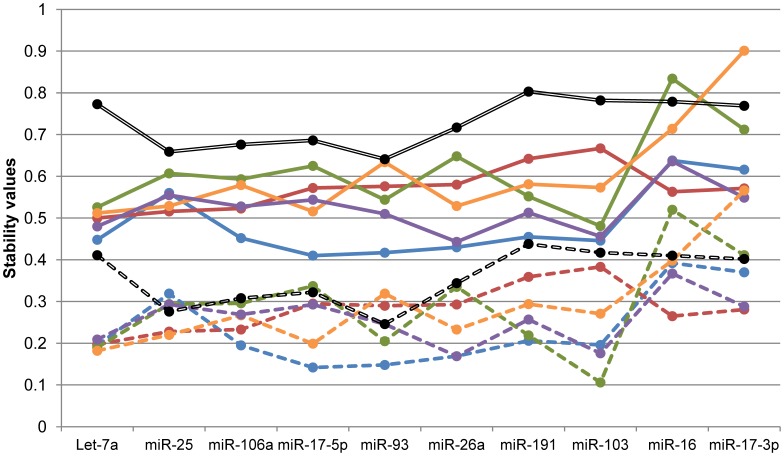
Stability values of each reference miRNA from geNorm^1^ and NormFinder^2^ algorithms. ^1^: [17], ^2^: [33]. Continuous lines: M-value from geNorm software; dashed lines: stability value from NormFinder software. Black lines: general study, blue lines: kidney, red lines: ovary, green lines: uterus, orange lines: skeletal muscle, purple lines: liver.

Comparing the stability values obtained in the entire data with those obtained in each tissue group, the M and SV values generally improved when tissues were treated as separated (Figure 2). This is due to the decreasing variability in the sample group analysed (Figure 2). There were some miRNAs which considerably improved their stability values when they were measured in a single tissue, such as Let-7a, miR-26a, miR-103 or miR-17-5p. These four miRNAs had discrete stability values in the entire data, but were the best stable miRNAs when data were divided by tissues. Thus, these miRNAs are very stable in a specific tissue, suggesting that the stability of miRNAs expression varies between tissues, and would be the best option for reference miRNAs if we are interested in an experimental design using only one tissue. However, they would not be the best option in multi-tissue experiments because their stability will decrease.

It is important to take into consideration the physiological status (pre and post-pubertal) of the sows because it is known that hormones could affect the gene expression. However, the ten reference miRNAs tested in this study showed no significant differences in miRNA expression between pre and post-pubertal sows. Then, it seems that the expression of these 10 miRNAs in the studied samples is stable under different hormonal environments.

### Determining the Optimal Number of Reference miRNAs

**Figure 3:**
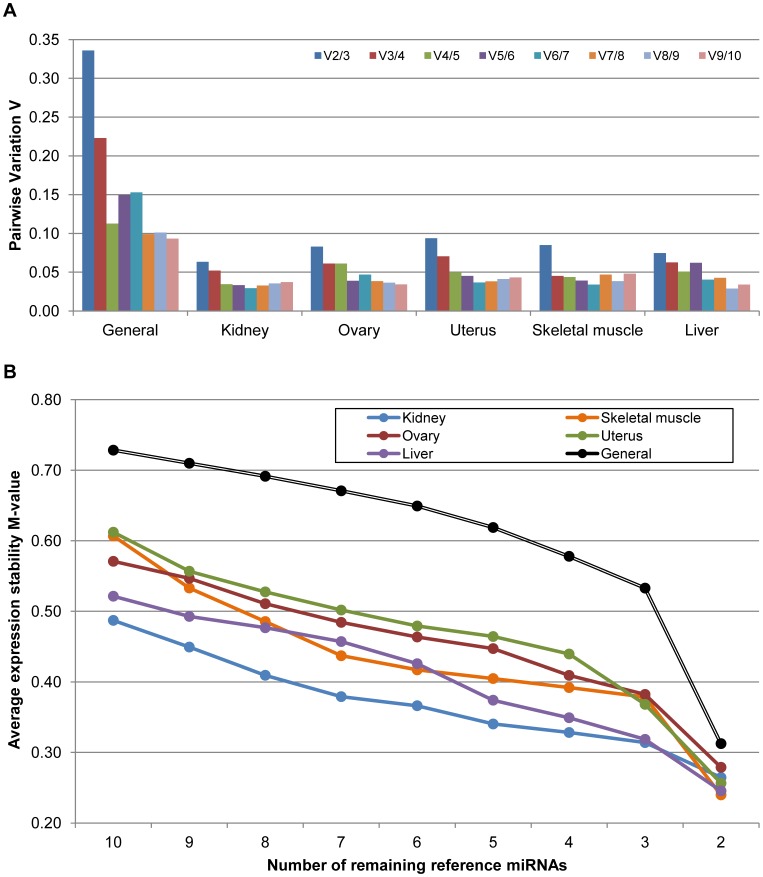
Variation of the miRNA stability expression. (A) Pairwise Variation between two sequential normalisation factors containing an increasing number of reference genes. Data were taken from all samples and dividing it by tissue origin. According to geNorm algorithm [17], a 0.15 cut-off was accepted. For the general study with many tissues it would be recommendable the use of 5 reference miRNAs. In the studies with a single tissue it would be enough to include 3 reference miRNAs. (B) Average expression stability M-values of remaining control genes during stepwise exclusion of the least stable reference miRNA. M-values were calculated by using geNorm algorithm [17]. Black line: general study, blue line: kidney, red line: ovary, green line: uterus, orange line: skeletal muscle, purple line: liver.

The optimal number and choice of reference miRNAs for qPCR data normalisation must be experimentally determined. Moreover, more than one reference miRNA should be used [32]. In order to determine the optimal number of reference miRNAs needed for a proper correction of RT-qPCR data, the pairwise variation between two sequential normalisation factors containing an increasing number of miRNAs were studied using the geNorm algorithm (V-values, Figure 3). A large variation means that the added gene has a significant effect and should preferably be included for calculation of a reliable normalisation factor [17]. Analysing the entire data, the lowest V-value was obtained using the ten miRNAs studied (Figure 3A). However, use of such a large number of reference miRNAs is unlikely due to experimental requirements and the economical costs. Following geNorm developer recommendations, taking a 0.15 cut-off value on pairwise variation could be enough for a reliable normalisation. In this sense, five reference miRNAs would be necessary for normalisation studies with multiple tissues (V-value = 0.11). Despite of the differences between the studies, these results could be in accordance with [20] and [22], recommending more than one reference miRNA in most situations and three reference miRNAs as the optimal number, respectively.

In studies considering only one tissue, the use of three reference miRNAs would be optimal, taking into account that the V-values using three reference miRNAs were below 0.10 in all tissues (Figure 3A). To contrast the results obtained, the average of stability M-value from geNorm in a stepwise exclusion of the least stable reference miRNA was calculated (Figure 3B). The necessity of including a third reference miRNA was evidenced taking into account the average expression stability variance from two reference miRNAs to three, even reaching five reference miRNAs in studies with multiple tissues in order to minimise the variation in the stability M-value. The high variation on the average expression stability M-values using only two reference miRNAs instead of three was evidenced. This variation became stable when a third or fourth reference miRNA was added. The increased variation in the M-value when a low-stability miRNA was used as an endogenous control was also proved. For example, in the last four miRNAs used in skeletal muscle, showing a high variation every time a reference miRNA was excluded, and the expression stability M-value became stable from the seventh to the third reference miRNA used. A similar situation happened in kidney tissue. Nevertheless, the expression stability M-value variation in the entire data remained constant using from the tenth reference miRNAs to the fifth, where it started to increase.

In conclusion, this work has evaluated the stability of ten miRNAs in different porcine tissues and breeds showing that they could be used as reference miRNAs. Stability values reflect that tissue is an important variability factor and it must be taken into consideration in the experimental design. It is recommended the use of five reference miRNAs: miR-93, miR-25, miR-106a, miR-17-5p and miR-26a in studies which include multiple tissues. For studies in a specific tissue, the optimal would be the use of three reference miRNAs which is sufficient to obtain a reliable normalisation of data. The most stable reference miRNAs vary between the tissues studied. In kidney it is recommended miR-17-5p, miR-93 and miR-26a. In ovary the best options are Let-7a, miR-25 and miR-106a, while in uterus we recommend to use miR-103, Let-7a and miR-93. If the study is focused in skeletal muscle, we encourage using Let-7a, miR-17-5p and miR-25, but if we are working with liver, the most stable miRNAs to be used as reference miRNAs are miR-26a, miR-103 and Let-7a.

Overall, this study provides valuable information about the porcine reference miRNAs that can be used in order to perform a proper normalisation when relative quantification studies are undertaken. Further experiments should be made to construct a database with recommended reference miRNAs for each tissue in porcine and also for multiple tissue studies.

## Materials and Methods

### Sample Collection

Samples were collected from five different pig tissues (skeletal muscle, ovary, uterus, kidney and liver). Pigs included in the study came from different breeds: Iberian (IB), Meishan (ME), Vietnamese (VT), Landrace (LD) and Large White (LW). Two samples per breed and per tissue were analysed (n = 50). All animals were females in different physiological state (per-pubertal: LD and LW; post-pubertal: IB, VT and ME) and age (6 month old: LD and LW; 1 year old: IB and VT; 2 year old: ME). All samples were taken from slaughterhouse (PRIMAYOR, Mollerussa, Spain) under veterinary supervision. Samples were immediately snap-frozen in liquid nitrogen and stored at -80°C until use.

### Candidate Reference miRNAs Selection

Ten miRNAs were selected to be evaluated as reference genes according to the literature: Ssc-let-7a, Ssc-miR-103, Ssc-miR-17-3p, Hsa-miR-25, Hsa-miR-93, Ssc-miR-106a, Ssc-miR-191, Ssc-miR-16, Ssc-miR-26a and Ssc-miR-17-5p [20], [22], [24], [26], [28]. Selection was done considering their stability values on the published studies.

### RNA Isolation and cDNA Synthesis

Total RNA was isolated using TRIzol® reagent following the manufacturer’s recommendations (Invitrogen, Carlsbad, USA). RNA was quantified by absorbance using ND 1000 Nanodrop® Spectrophotometer (Thermo Scientific, Wilmington, USA) and checked for integrity by using RNA 600 Nano kits (Agilent Technologies, Santa Clara, USA) on an Agilent 2100 Bioanalyzer.

Reverse transcription (RT) reactions were performed in duplicate using total RNA as previously described [34]. Briefly, 600 ng of total RNA in a final volume of 20 µL including 2 µL of 10x poly(A) polymerase buffer, 0.1 mM of ATP, 0.1 mM of each dNTP, 1 µM of RT-primer, 200 U of M-MuLV reverse transcriptase (New England Biolabs, USA) and 2 U of poly(A) polimerase (New England Biolabs, USA) was incubated at 42°C for 1 hour and 95°C for 5 minutes for enzyme inactivation. Minus reverse transcription (RT) and minus poly(A) polymerase controls for each tissue were included.

### Quantitative Real-time PCR Reaction

Quantitative PCR reactions were performed in a final volume of 20 µL including 10 µL of FastStart Universal SYBR Green Master (Roche, Germany), 250 nM of each primer (with the exception of Ssc-let-7a and Hsa-miR-93, with 125 and 200 nM, respectively) and 5 µL of a 1∶2000 dilution of the cDNA (except for Ssc-miR-17-3p where a dilution of 1∶200 was used). See Table 2.

Standard curves were generated in order to calculate the RT-qPCR efficiency. All standard curves were done by using 10 fold serial dilutions from a pool of cDNA of all the samples (n = 50) and were included per duplicate in all qPCR assays. For Ssc-miR-17-3p, a 2-fold dilution standard curve was used. Reactions were incubated in a 96-well plate at 95°C for 10 min, followed by 40 cycles of 95°C for 15 sec and 60°C for 1 min on a 7900HT Real-Time PCR System with 7900HT SDS v2.4 (Applied Biosystems). DNA primers for each miRNA were designed following the methodology described by [34] (Table 1).

Measurements were performed in duplicate. Minus RT controls, minus poly(A) polymerase controls and no template controls were included. Moreover, melting curve analysis was performed in each assay in order to detect unspecific amplifications.

### Stability Expression Analyses

Quantities from each sample were obtained from the calibration (standard) curve added in each RT-qPCR reaction. Stability of each candidate miRNA was tested using geNorm v.3.5 algorithm [17] and NormFinder algorithm [33]. The geNorm algorithm calculates the gene expression stability M Value for each candidate reference gene based on the average pairwise variation between all studied genes. NormFinder is based on an ANOVA mathematical model and estimates intra- and intergroup variation providing the best stable candidate reference gene and also the best stable pair of them taking into account the subgroups in which data is distributed. In both programs, the lowest stability values indicate the most stably expressed reference genes allowing them to rank according to their expression stability.

RT-qPCR expression data was also analysed by a two-way analysis of variance (ANOVA) with the General Linear Models procedure of the Statistical Package for the Social Scientists (IBM® SPSS® Statistics 19; IBM Corporation, Armonk, USA). The model used included breed and tissue as fixed factors and breed by tissue interaction. GeNorm v.3.5 software [17] was used to obtain the normalization factor (NF), necessary to normalize each sample quantity obtained from qPCR reaction. Next, fold changes were calculated in relation to the highest sample normalized value for each miRNA. Fold changes were log_2_ transformed and significance threshold was set at α<0.05. Student-Newman-Keuls (SNK) and Scheffe tests were used to determine significant differential expression between breed or tissue groups.
